# A Semiotic Cultural Psychology Analysis of Musical Improvisation: Creativity as an Affective-Sociocultural Act

**DOI:** 10.1007/s12124-026-10026-z

**Published:** 2026-09-07

**Authors:** Diogo Monzo, Vlad P. Glăveanu

**Affiliations:** 1 Institute of Psychology, University of Brasília, Brasília, Brazil; 2 DCU Business School, Dublin City University, Dublin, Ireland

**Keywords:** Creativity, Affective-Semiotic Regulation, Distributed Creativity, Cultural Psychology, Musical Improvisation, Developmental Processes

## Abstract

This article examines the role of Affectivity in collaborative musical improvisation from a cultural-semiotic perspective. Drawing on published empirical accounts of free and jazz improvisation, we analyze how affective-semiotic regulation participates in the changing relations among musicians, musical materials, perspectives, and possibilities for action. The analysis focuses on pleromatization and schematization, perspectival-reflective semiosis, self-regulation, and orientation toward future possibilities. We argue that Affectivity operates across the distributed relations described by the 5 A’s framework, participating in how these relations acquire significance and orient creative action. On this basis, the 6 A’s Model is proposed as a heuristic extension that makes this transversal regulatory dimension explicit. The article contributes an affective-semiotic account of how creative activity can remain organized while preserving openness to possibilities that are not determined in advance.

## Introduction

In my initial exchanges with Vlad Glăveanu, we began discussing the role of affect in creative processes. I shared an episode with him that was deeply significant to me. In 2015, during a concert in Hungary, a series of unforeseen events occurring just before the performance left me gripped by nervousness and anxiety while I waited in the dressing room to go on stage. Once the concert began and I was on stage, my hands and feet were trembling so intensely that playing seemed impossible. I found myself facing a massive challenge, as the rest of the concert—and its continuation—hinged on my ability to regain emotional stability.

That was when a phrase from my college piano teacher echoed in my mind: “When you’re nervous, try playing as if you were in your own living room.” I looked inward and focused on accessing memories of my home studio back in Brazil. I mentally transported myself to that space—a place steeped in memories, gestures, and the emotional significance accumulated over a lifetime—where I feel safe and grounded. Gradually, my perception of the situation shifted; what had initially seemed intimidating and restrictive became familiar and calm, allowing me to reconnect with the music and with the very reason I want to be a musician every day.

This episode is significant not only because it demonstrates the transition from one emotional state to another, but also because it poses a broader question about creative action: How can individuals maintain engagement when the conditions for action become uncertain, unstable, or affectively challenging? The concerto itself did not undergo significant changes, nor did the technical demands of the piano performance. What changed was how I perceived, imagined, and executed the work in the face of instability. This episode invites us to reflect that creativity is not limited to generating novel results; the creative path toward novelty requires the actor (or creative agent) to sustain and reorganize their own potential for action as experiences unfold.

This issue resonates with sociocultural and distributed approaches to creativity. Such approaches challenge the notion that creativity resides exclusively within the individual’s mind by conceptualizing creative action as the product of interactions among people, cultural histories, material environments, symbolic resources, and opportunities for action (Cole & Engeström, 1993; Glăveanu, 2014, 2015; Hutchins, 2000). This understanding is not unique to these approaches; in music research, similar perspectives understand creativity as a process distributed among musicians, instruments, social and material environments, histories of interaction, and their dynamic relationships (Clarke & Doffman, 2017; van der Schyff et al., 2018). In this sense, what a person creates cannot be dissociated from the social and material world in which the action occurs. Glăveanu’s “5 As” model articulates this relational orientation by conceptualizing creativity through the dynamic interrelationships among Actor, Action, Artifact, Audience, and Affordance.

Yet describing the distributed relations that constitute creative activity leaves another question open: How are these relations experienced and regulated as creative action unfolds? Embodied, enactive, and ecological approaches to musical creativity have advanced understanding of how musicians adaptively engage with social, material, and sonic environments (Schiavio et al., 2024; van der Schyff et al., 2018). The question pursued here is more specific: How does the changing affective significance of these relations participate in sustaining or redirecting creative action over time? Actors do not encounter audiences, artifacts, or affordances from an experientially neutral position. What becomes salient, threatening, inviting, or possible changes over the course of action. Engagement can intensify or diminish; uncertainty can become restrictive or generative; the gesture of another person can interrupt an emerging direction or open an unexpected one. Understanding how creativity is distributed also requires attention to how people sustain and reorganize their engagement within these changing relations.

Cultural psychology serves as a theoretical basis for analyzing this process. In Valsiner, 2014, Valsiner, 2021, Valsiner, 2024, affectivity is not treated simply as an emotional response added to an otherwise cognitive process. Affect participates in how situations acquire significance and how people orient themselves toward what is happening and toward what might happen next. Through affective-semiotic processes, lived experience is structured in ways that may limit certain possibilities while enabling others. In this context, self-regulation does not denote an isolated individual managing internal states; it operates through the interplay among self, others, cultural meanings, material conditions, and anticipated possibilities.

Musical improvisation offers a valuable context for examining these dynamics. Improvisers operate in an environment of unpredictability and uncertainty where they cannot know exactly what will come next, making it impossible to maintain absolute control over actions. They interact within a continuous flow of sonic dialogues—listening and responding to one another, negotiating possibilities and moments of resistance, and simultaneously evaluating the emerging musical material—while continuously adjusting their actions as the performance progresses. This scenario allows us to observe that the creative process depends not only on the coordination among agents, actions, artifacts, the audience, and affordances, but also on the musicians’ ability to remain engaged as these relationships evolve.

Against this background, this article asks: How can an affective-semiotic perspective clarify the regulatory dynamics that sustain and reorganize distributed creative action during musical improvisation? Drawing on a purposively selected set of published empirical accounts of collaborative musical improvisation, we examine how affective-semiotic organization participates in musicians’ meaning-making and in how they perceive, evaluate, and respond to evolving creative situations. Particular attention is given to dynamics involving moments of uncertainty, listening and response, trust, tension, shifts in perspective, engagement with instruments and sound materials, anticipation, and possibilities for transformative action.

Hence, the article views affectivity as a regulatory dimension of the creative process. Our analysis discusses how an affective-semiotic perspective can expand the “5 As” framework—supporting an extension of the model to “6 As”—as a heuristic for examining how affectivity participates in distributed creative processes.

## Affectivity in the Dynamics of Distributed Creativity

Although our interest in affectivity is grounded in this relational understanding of creativity, we are not questioning whether distributed approaches have neglected the social, material, or experiential dimensions of creative activity. We aim to explore possibilities for understanding the relationships among Actor, Action, Artifact, Audience, and Affordance, acknowledging that these elements do not possess fixed meanings. This characteristic makes the process particularly interesting and calls for two necessary reflections: (1) How each creative agent perceives, feels, and assigns importance to these elements, and (2) How the relationships between them may shift throughout the process.

In cultural semiotic psychology, affectivity plays a central role. More than mere emotional responses to external events, affects are understood as a fundamental dimension of human developmental processes. Through them, human beings not only orient themselves regarding ongoing and potential future events but also attribute meaning to lived experiences (Valsiner, 2014, 2024). Affective-semiotic self-regulation processes do not determine actions; rather, they contribute to the organization of experience by participating in the field where possibilities for action take on different meanings for the individual over time. The same applies to creativity. During the creative process, the actor (or creative agent) can, through creative action, generate innovations and alter the course of an event along its trajectory. This reality helps us understand that aspects of a situation, such as those appearing significant, inviting, threatening, familiar, uncertain, or worth pursuing, can change as the context evolves.

From this perspective, affective-semiotic regulation refers to the processes by which individuals organize and reorganize their relationship to evolving situations through signs and affective meanings. This form of regulation does not require that experience be fully represented or verbally articulated prior to action. Signs may temporarily stabilize experience, orient action, and distinguish possibilities within situations that are partially ambiguous or affectively diffuse. At the same time, these organizations remain open to transformation as new events emerge.

This conceptualization also necessitates careful consideration of the notion of self-regulation. The “self,” as used here, does not refer to an isolated individual managing an internal emotional state. Another improviser’s gesture, sonic dialogues, an instrument’s possibilities or resistances, an audience’s response, a remembered experience, or an anticipated musical direction can reorganize a musician’s orientation. Self-regulation is understood as a relational process that involves reorganizing the individual’s orientation within a field that is simultaneously social, material, cultural, and affectively significant.

An affective-semiotic approach does not supplant distributed, embodied, enactive, or ecological accounts of musical creativity. It focuses on how the relationships described by these approaches become significant for individuals and how changes in this process affect subsequent possibilities. This temporal aspect is particularly salient in improvisation, where musicians must continue to act even as the meaning and consequences of the situation remain partly unsettled.

Phenomenological research on musical absorption similarly examines transformations in attention, bodily awareness, agency, and relations between self and environment during musical engagement (Høffding, 2019; Vroegh, 2019). The present approach is complementary but addresses a different question: How experiences acquire changing significance through semiotic mediation and how this participates in regulating subsequent creative action. This distinction clarifies the specific contribution of an affective-semiotic perspective without positioning it as an alternative to phenomenological or enactive accounts.

The integration of Affectivity into the 5 A’s framework should not be regarded merely as the addition of an independent component. Affectivity operates across the developing relationships among Actor, Action, Artifact, Audience, and Affordance. For example, an affordance may not retain the same significance for an actor throughout a creative process; an audience may become encouraging, demanding, or disruptive; and an artifact may enable certain possibilities while constraining others. Affectivity highlights the shifting experiential significance of the relations already described by the 5 A’s. At this stage, we leave open the question of how Affectivity should be positioned within this framework and proceed to examine the dynamics through which these relations come to matter and are reorganized during creative activity.

## Collaborative Musical Improvisation and Empirical Sources

Collaborative musical improvisation refers to musical practices in which two or more musicians participate in creating a performance whose course is not fully determined in advance. The term does not designate a specific genre or tradition of improvisation. The phenomena analyzed include both free improvisation and jazz improvisation, acknowledging that each of these practices possesses its own histories, conventions, and forms of organization. For this study, these practices are linked by the relational and dialogic nature of musical creation; that is, musicians act and orient themselves within a flow continuously shaped by their own actions and the actions of others.

This understanding draws upon the concept of Distributed Free Improvisation (Monzo, 2025), which situates musical creation within an extensive network of relations involving individuals, materials, and the broader social and cultural contexts they inhabit. From a sociogenetic perspective, these relations are integral to the creative process, as musicians engage with them as cultural subjects whose modes of action, interpretation, and interaction have developed throughout their life trajectories. Affectivity plays a central role in this process. Monzo (2025) conceptualizes affective-semiotic self-regulation as foundational to musicians’ dialogical interactions, permeating the relationships among Self, Other, and World, and regulating the meanings that guide creative action. This relational perspective can be extended to collaborative musical improvisation more generally. A musical gesture is linked not only to another musician’s gesture but also to instruments, sonic materials, prior experiences, expectations, shared or divergent understandings of the situation, and the cultural resources musicians contribute. Collaboration encompasses more than interpersonal coordination. It offers a particularly valuable context for examining how individuals orient themselves within a dynamic field of social, material, cultural, and affective-semiotic relations.

This article presents a theoretically informed interpretive analysis of published empirical accounts of collaborative musical improvisation. We selected sources and episodes that illuminate shifts in affective orientation, meaning, and possibilities for action, including moments involving uncertainty, active listening, tension, changes in perspective, engagement with instruments and sound materials, and orientation toward future possibilities. We interpreted these episodes through the affective-semiotic concepts developed in the preceding sections.

The materials come from Menezes (2011), Monzo (2025), Wilson and MacDonald (2017), Monson (1996), and Bailey (1993). They bring together different forms of collaborative musical improvisation and different ways of gaining access to musicians’ experiences and interactions. Menezes provides qualitative accounts from three experienced improvisers, gathered through individual interviews and a retrospective verbal protocol in which the musicians watched and commented on a recording of their performance. Monzo (2025) contributes empirical material from distributed free improvisation, including musicians’ accounts of interaction, listening, shifts in relational positioning, and attempts to articulate affectively complex musical experiences. Wilson and MacDonald provide accounts from group free improvisation, with particular attention to musical choices, uncertainty, trust, and differences in how musicians interpret and make sense of a shared musical situation. Monson’s analyses of jazz performance provide detailed accounts of interaction among improvisers, including musical exchanges, temporal coordination, and the ways musicians respond when established patterns of coordination are disrupted. Bailey’s conversations with improvising musicians offer first-person reflections on form, anticipation, musical possibilities, and the relation between previous experience and what may be created in performance.

The materials under consideration possess distinct methodological histories, and this diversity informs the approach taken in this analysis. They do not constitute a unified dataset and are not regarded as equivalent forms of evidence. Each case remains anchored to the context of its original production and interpretation. The analysis focuses on what these documented moments reveal when examined through an affective-semiotic perspective. These materials serve a theoretically generative function, offering situated encounters through which concepts may be examined, differentiated, and, when appropriate, reconsidered.

Synthesizing these situations enables movement between various scales of the creative process. At times, attention focuses on a musician’s lived experience; at other moments, it shifts to exchanges between improvisers, changes in sonic material, an instrument’s possibilities, and the direction the musical performance is taking. Tracing these transitions is essential for the argument presented in this study. Affectivity emerges not as an attribute confined to the musician, but through how relationships gain significance and contribute to the ongoing organization of creative action.

## Affectivity as Lived Orientation

If “art is the social within us” (Vygotsky, 1971, p. 249), we must recognize that affectivity is a key part of what makes us social. (Monzo, 2025). Human experience is inherently responsive to environmental stimuli. Certain encounters capture attention, while others provoke hesitation or resistance; specific sounds, gestures, objects, or memories may carry particular significance, whereas others remain peripheral. Artistic activity renders these distinctions especially apparent. Individuals may be moved, oriented, unsettled, or attracted by an experience without being able to articulate precisely what is felt or why it holds significance.

In this article, Affectivity refers to the qualitative, embodied, and valuative dimension of experience through which situations, relations, objects, and possibilities acquire personal significance and participate in orienting action. Affectivity encompasses recognizable emotions and feelings, as well as more diffuse qualities, tensions, attractions, hesitations, and orientations that may remain partially differentiated or not fully articulated verbally. Affectivity addresses how individuals experience situations as meaningful and how certain aspects of those situations achieve greater experiential significance than others. Musical experience cannot be reduced to a stimulus eliciting a measurable response; what matters is how sounds assume different meanings within individuals’ concrete lifeworlds (Allesch, 2016).

A semiotic foundation for the qualitative dimension of this experience can be found in Peirce’s account of Firstness. Peirce describes qualities of feeling as qualities considered in themselves, distinguishing them from the concrete event of experiencing them (Peirce, 1931–1958, CP 1.304). His definition of feeling is equally significant. Feeling involves neither analysis nor comparison and has a positive qualitative character that is what it is in itself, regardless of anything else (Peirce, 1931–1958, CP 1.306). Firstness offers a conceptual basis for recognizing a qualitative dimension of experience without reducing it to an already identified emotion or conceptual judgment. It should not be equated with Affectivity itself. Its relevance here lies in acknowledging qualities that can be lived without being clearly differentiated.

Peirce’s broader semiotic account also connects feeling with the formation of orientation. A qualitative experience need not remain isolated from processes of signification. He allows a presentment to function as a sign, showing how sensory qualities such as tones, colors, and odors can enter relations through which meaning is constituted (Peirce, 1931–1958, CP 1.313). His discussion of belief adds another dimension to this relation. As Klempe (2022) notes in his reading of Peirce, feelings associated with doubt and belief participate in inquiry, the formation of conviction, action, and the establishment of habits. Feeling is implicated in how something is experienced and in the force with which certain orientations are sustained.

Affectivity does not necessarily take the form of clearly differentiated or verbally articulated feelings. Lehmann and Valsiner (2017) emphasize the depth and indeterminacy of affective experience, noting that intense affective processes may remain undifferentiated and difficult to put into words. The embodied character of this orientation is equally important. Maiese (2014) approaches emotion through an enactive perspective, emphasizing the close relation between bodily processes, cognition, and a person’s engagement with the environment. Her notion of affective framing is particularly relevant here because it draws attention to how situations are encountered through embodied patterns of concern and evaluation. Through affective framing, affectivity participates in what becomes salient and significant within a situation and in the possibilities toward which a person becomes oriented. This embodied dimension also has a longer history in psychological thought. Klempe’s (2025) discussion of Kant shows how bodily states of pleasure and displeasure can be experienced without immediately acquiring a precise conceptual form. This distinction is relevant to our argument because it cautions against identifying affective experience with the vocabulary available to describe it. What a person can name is one way in which an affectively charged experience becomes articulated.

Valsiner’s cultural psychology serves as a theoretical lens for understanding how such qualities become part of personal meaning and regulation. Within this perspective, affectivity can take different degrees of semiotic differentiation. Valsiner’s hierarchy of affective semiosis ranges from physiological activation and immediate experience to specific emotion terms, generalized feelings, and hyper-generalized affective-semiotic fields (Valsiner, 2014). Some of these significant forms are difficult to put into words or conceptualize; they operate within the realm of pre-reflective experience, yet they exert a strong regulatory force on action, guiding how the individual relates to a situation without taking on a precise conceptual form.

His theory of dynamic semiosis (Valsiner, 2024) sharpens this point by distinguishing point-like and field-like signs. Words and relatively delimited categories typically take point-like forms, whereas musical and visual experiences may take field-like forms with less sharply defined boundaries. The distinction is particularly valuable for music because an experience does not need to become a discrete verbal category to be meaningful. In the preface to Cultural Psychology of Musical Experience, Valsiner (2016) describes musical meaning as proceeding through affective mechanisms and a pleromatic pathway toward generalized fields of feeling, observing how difficult it can be to speak about music compared with living its affective quality. Musical experience can thus make perceptible forms of significance that exceed the conceptual precision of language. A verbal account can be approached as one possible articulation of lived experience, without assuming that it transparently reproduces that experience.

Affectivity possesses regulatory force. Meaning extends beyond the representation of past events. Affective semiosis can intensify, inhibit, sustain, or redirect potential courses of action. Valsiner identifies dynamic hierarchies of signs as resources for self-regulation, enabling individuals to organize experience and orient action. This regulatory aspect clarifies why two elements that seem equally available within a situation may not hold equal significance for an individual. One element may attract attention while another recedes; one possibility may be pursued, whereas another is avoided or remains unexplored.

Collaborative musical improvisation makes these processes especially apparent. Musicians engage in contexts where sounds, gestures, silences, instruments, memories, expectations, and others’ actions may take on varying significance from moment to moment. For example, a gesture may invite participation, while a silence may be experienced as openness, hesitation, or tension. An unexpected sound may function as a disturbance, a source of curiosity, or the initiation of a new musical direction. The significance of these events cannot be determined solely by their acoustic properties. Instead, meaning is constituted through the musician’s relationship to the situation, which encompasses bodily orientation, prior experience, cultural resources, interpersonal dynamics, and anticipated possibilities.

Affectivity addresses a question that a description of distributed relations alone leaves unresolved: How do specific relations become meaningful for participants in creative activity? People, instruments, sounds, environments, and possibilities all participate in creative situations, yet their significance for those involved is neither equivalent nor fixed. Affectivity highlights the qualitative, valuative, and regulatory dimensions of lived relations.

A subsequent analytical question concerns how these shifting significances are organized within experience and reach sufficient stability or intensity to guide creative action. The concept of affective-semiotic fields provides a framework for examining this process.

### Affective-Semiotic Fields

In an improvisation, not everything that is played carries the same weight. Certain sounds may pass almost unnoticed, while another seems to remain in the room, asking to be followed. A gesture that initially appears out of place may subsequently initiate a new musical trajectory. The transformation lies not necessarily in the sound itself, but in the position it assumes within the musician’s experiential framework. These small transformations show how creative activity is organized through differences in significance. Some elements become prominent, others recede, and previously peripheral aspects may acquire the capacity to influence subsequent developments.

Semiotic cultural psychology understands human psychological life as a continuous, affectively charged process of meaning-making that unfolds in irreversible time. In this framework, verbal and non-verbal signs actively regulate experience rather than merely represent it (Valsiner, 2014, 2021). Meaning arises through the co-genetic relationship between action and signification, so that phenomena and their contexts are constructed simultaneously within processual activity (Herbst, 1995; Valsiner, 2014). Within this dynamic, individuals organize action, emotion, and expectation by drawing on traces of the past while orienting themselves toward the future. This process semiotically extends the present to sustain experiential coherence. The human psyche is understood as an open, hierarchically organized system structured by interconnected layers of affective and semiotic processes (Valsiner, 2014).

Jaan Valsiner’s affective-semiotic model explains how emotions, meanings, and actions are organized through layered systems that integrate bodily dynamics, cultural processes, and symbolic mediation. Rooted in cultural psychology and semiotic theory, the model conceptualizes affect as a dynamic form of meaning-making, constituted and regulated by signs at varying levels of generalization. These hierarchically structured levels connect immediate physiological responses to more generalized, socially shared affective patterns, elucidating how individual experiences become embedded within values, cultural narratives, and normatively guided actions. The model examines continuities and transformations in affective life—the affective-semiotic organization of experience—by considering both individual processes and their cultural constitution.

**Fig. 1:**
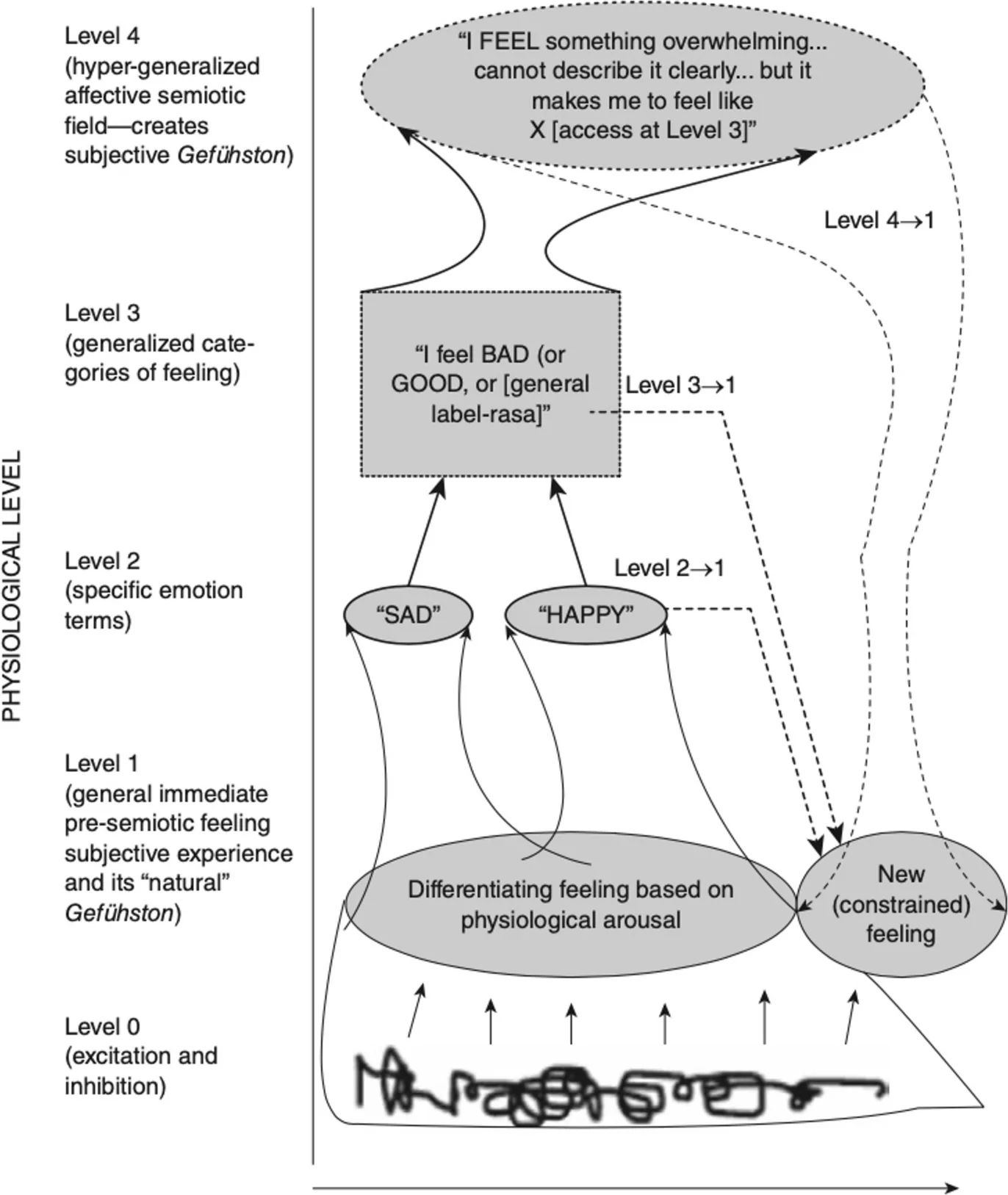
Generalization of signs: How affect operates (Valsiner, 2014, p. 126)

Figure 1 illustrates this process of sign generalization and affective regulation, showing how affect operates at different levels of semiotic mediation within the human psyche.

The model conceives affect as structured through a hierarchy of semiotic mediation levels. At Level 0, affect manifests as basic physiological activation and inhibition processes that precede conscious emotional experience. Level 1 represents an immediate, pre-semiotic feeling state, characterized by a vague and holistic sense of “feeling something” that is difficult to articulate verbally. At Level 2, this undifferentiated feeling is categorized using culturally available emotion labels, such as fear, sadness, or happiness. Level 3 encompasses the development of more generalized and moralized affective categories, including evaluative distinctions such as good/bad, right/wrong, and us/them, which are frequently employed in social communication and collective discourse. Level 4 denotes a hyper-generalized affective field that is largely non-verbalizable and functions as an overarching emotional atmosphere, orienting thought and action without explicit linguistic mediation.

Central to this organization are affective-semiotic fields, understood as semi-structured psychological regions characterized by blurred and permeable boundaries (Branco, 2021). These fields take shape and develop throughout the life course as individuals engage with recurring experiences, relationships, and cultural meanings (Valsiner, 2014, 2021). Over time, they may gain or lose strength, transform, or stabilize, depending on how they are affectively energized. As they become progressively generalized, such fields exert an increasingly powerful regulatory force, orienting values, beliefs, and modes of relating to self, others, and the world (Branco, 2016, 2023).

Collaborative musical improvisation gives a valuable context for examining these movements. Sounds, gestures, silences, and actions do not hold equal significance within the musician’s experience. Their affective weight can change as the musical situation develops, influencing what draws attention, what is maintained, and what emerges as a potential direction. Menezes’ Study 2, based on qualitative data from three experienced improvisers—PC, BP, and MM—provides an illustrative example. This study analyzed the musicians’ accounts through individual interviews and a retrospective verbal protocol, in which participants viewed a recording of their performance and commented on specific moments.

One of the creative processes identified in Study 2 was reiteration. Menezes describes it as a process associated with the creation of tension and musical personality. PC and MM expressed this in particularly concise terms:

"[musical] ideas become interesting through reiteration” (PC)

"if you do something during 30 seconds it is boring, but if you keep it for 4 minutes it grows a personality" (MM). (Menezes, 2011, pp. 56–57).

MM’s account points to a subtle transformation in the relation between musician and musical material. What is first experienced as “boring” does not remain affectively neutral simply because the musical idea continues to be repeated. Through reiteration, the same material comes to occupy another place in experience: it “grows a personality.” PC describes a similar movement when he says that musical ideas “become interesting” through reiteration. In both accounts, significance is constituted through the history of engagement with the material.

This movement can be understood in terms of affective-semiotic fields. The musical idea enters a field in which its significance is neither fixed nor contained exclusively in its sonic properties. As the musician remains with it, its affective weight can change. An aspect of the situation initially experienced as uninteresting may acquire enough significance to sustain attention, invite exploration, and orient subsequent action. The field functions as a regulatory mechanism by making certain possibilities more compelling than others. Klempe’s (2022) interpretation of Peirce elucidates the regulatory movement underlying this process. Feelings related to doubt and belief contribute to inquiry, action, and habit formation, connecting the experience of a situation to the intensity with which specific orientations are maintained. Here, the changing significance of the reiterated material participates in sustaining the musician’s orientation toward its further exploration. Windsor’s (2004) ecological approach to semiotics helps clarify this relation by situating signification within organism–environment relations and understanding affordances as possibilities for action that arise within these relations. In the present case, reiteration alters both the musician’s relationship to the musical material and the possible directions for action that the material presents.

This connection also positions Affectivity in direct relation to the distributed architecture of the 5 A’s (Glăveanu, 2015). Musical material may be understood as an Artifact, while its reiteration and transformation pertain to Action, and the possibilities it presents relate to Affordance. Yet the mere existence of an affordance does not fully account for why a musician chooses one possibility over another. The accounts of PC and MM indicate that the significance of these relationships can evolve through experience. Affectivity helps us describe this changing experiential weight. An affordance that initially holds minimal appeal may gradually grow in significance until it offers a viable direction. Allesch’s (2016) cultural-psychological approach to musical experience emphasizes that sounds assume meaning within the individual’s concrete lifeworld. Musical material, then, does not carry significance on its own; its significance depends on how it enters into the musician’s experience.

A second group of accounts brings the interpersonal dimension of this regulation into view. Menezes notes that the musicians repeatedly described improvisation through the metaphor of conversation, using terms such as talk, language, dialogue, and conversation:

“that’s like the flow of a conversation” (BP)

“[the error] is solved and the conversation goes on…” (PC)

"I try to understand what [the other musician, during improvisation] is talking about…” (MM)

“What I want with my language…” (BP). (Menezes, 2011, p. 57)

The concept of error is particularly significant in this context. Menezes reports that the musicians identified technical flaws, instances of playing without engaging with the musical context, and what MM described as a “disruption of musical unity” (Menezes, 2011, p. 57) as distinct forms of error. At the same time, all three participants regarded errors as potential sources of creativity, suggesting that resolving these errors could stimulate further creative processes.

Seen this way, an error does not necessarily produce a predetermined experiential outcome within the musical situation. It may disrupt an established relationship, require focused attention, or acquire new significance depending on the musicians’ subsequent responses. PC’s phrase, “the conversation goes on,” effectively encapsulates this regulatory process. An event occurs that necessitates adjustment, yet the interaction remains open to further development. The critical factor is how the event is integrated into the wider field of significance that informs the musicians’ subsequent actions.

MM’s attempt to “understand what the other musician is talking about” adds another dimension to this process. Listening demands an orientation toward another individual whose actions affect the field of musical possibilities. Schiavio et al.’s (2024) enactive perspective helps us understand this relation in terms of meaning-making through embodied and situated interaction. Affectivity participates in how these actions are lived—as invitation, disruption, curiosity, resistance, continuity, or as qualities that may not yet have a precise verbal name.

Seen from the 5 A’s framework, Affectivity is integrated with Actor, Action, Artifact, Audience, and Affordance. This integration facilitates analysis of how experiential significance develops through relationships among these elements. For example, an Artifact may gain importance through repeated use, while an Action by another musician may transition from being disruptive to enabling further collaboration. Similarly, an Affordance may be perceived as attractive, uncomfortable, or irrelevant depending on the contextual field. The distributed organization of creativity is also an organization of significance. Affective-semiotic fields provide a way of examining how this significance becomes sufficiently powerful to orient creative action toward some possibilities while others remain in the background.

### Pleromatization ↔ Schematization

Affective experience is not always easy to define. Sometimes, what we feel or sense as important goes beyond the categories we use to describe it. Valsiner (2006, 2014, 2021, 2024) distinguishes two analytically different yet inseparable processes of signification, pleromatization and schematization. These should not be understood as sequential stages. They describe interdependent movements through which experience can vary in its degree and form of semiotic articulation in irreversible time. Every act of sign formation involves abstraction; however, this abstraction remains connected to the experiential field from which it develops (Valsiner, 2006). Meaning does not originate only in explicit concepts or language. It can take shape through what Valsiner (2024) describes as a vague “aboutness,” an orientation toward experience that has significance before reaching greater symbolic or conceptual differentiation. Pleromatization and schematization allow us to examine movements between relatively diffuse, field-like forms of meaning and more differentiated forms of articulation without treating one as simply replacing the other.

Schematized signs are products of abstraction, reducing the richness and complexity of experience through generalized categories. They operate through verbal signs, categories, and logical schemes that provide experience with more differentiated and abstract forms of semiotic organization (Valsiner, 2014). This process resonates with Peirce’s account of Thirdness, particularly his association of representation with mediation and generality (CP 1.337–1.340). Without equating schematization with Thirdness, this connection highlights how particular experiences can be mediated through more general forms of signification. Schematization supports the organization of experience through relatively stable categorical distinctions while remaining dynamically interconnected with pleromatization (Valsiner, 2006). Such stabilization selects from the complexity of lived experience. What becomes articulated in a word, category, rule, or musical convention does not reproduce the experiential field in its entirety. It gives certain aspects greater definition while others remain less differentiated. Schematization can make aspects of experience available for communication, reflection, and orienting action. This limitation becomes particularly apparent in experiences involving values, aesthetic judgments, and existential orientations, whose experiential richness may resist complete categorical articulation (Klempe & Lehmann, 2021).

Pleromatization describes a different movement within this relation. It preserves or expands the field-like quality of meaning, allowing experiential relations to remain affectively connected without requiring their immediate differentiation into discrete units (Valsiner, 2024). Its relevance lies less in identifying a particular kind of feeling than in attending to forms of significance that can be intensely lived without being fully articulated in discursive terms (Innis, 2020). Music is particularly revealing in this respect because sonic experience can hold affective significance without being immediately translated into linguistic categories (Monzo and Branco, 2026). Pleromatization and schematization can participate in the same experiential movement as a person lives a situation through partially differentiated qualities while simultaneously finding signs through which aspects of that experience acquire a more delimited form.

This relationship is particularly noteworthy when musicians attempt to articulate experiences that are difficult to express verbally. An illustrative example is found in Monzo’s (2025) microgenetic analysis. In this instance, P2 recounts an experience during distributed free improvisation, incorporating musical interaction, the sound of the piano, and aspects of his physical environment into his description of the moment.

I remember a time when I was so integrated with… we were so sunk into it, into the proposal, then I started like this, just here… my studio is a little bit […] you can see that here there’s a light beam coming through the window. So, I just stood here looking over there and stuff. Seriously, I really let myself go. I looked at the view here and I enjoyed it. This feeling is something that… that the great sound mass of the piano provides for you […] Yeah… it was more, I don’t know what the right word is for it… I was levitating (P2, Interview, 184–198, as cited in Monzo, 2025, p. 73).

What is striking in P2’s account is not the presence of any single identifiable emotion. His description moves across relations. He speaks of being “integrated” and “sunk into” the proposal, then turns toward the studio, the light entering through the window, the view, and the “great sound mass of the piano.” These elements appear together in his reconstruction of the moment, making it difficult to isolate the musical experience from the social, material, perceptual, and affective relations through which it was lived. The experiential field thus extends beyond the sounds produced, encompassing the environment in which those sounds gain significance.

This discussion is particularly relevant to the distributed conception of creativity advanced in this article. Light and view should not be regarded merely as external circumstances that surround an otherwise exclusively musical process. They enter P2’s account as part of the situation in which his orientation changes. Likewise, the piano is not described only as an instrument upon which an action is performed. P2 speaks of something that the “great sound mass of the piano provides for you.” The formulation places the musician in relation to sonic and material qualities that shape how the experience is organized. From the perspective outlined in the previous section, the significance of Actor, Artifact, Action, and Affordance is inseparable from the changing affective weight of these relationships as the situation unfolds.

The expression “great sound mass of the piano” is particularly evocative. Our analytical focus is on what P2 is doing when he uses this expression, without treating the sound mass itself as a pleromatic sign. “Mass” does not identify a discrete acoustic parameter or specify a particular musical structure. It gives a certain form to an experience of sound through an image of density and extension. An aspect of the sonic experience becomes articulable, while its meaning remains relatively open. The expression situates the listener within the dynamic between experiential richness and semiotic differentiation, as described by Valsiner through the concepts of pleromatization and schematization.

This movement becomes more explicit when P2 reaches the end of the account and hesitates, “I don’t know what the right word is for it.” This hesitation is significant because it reveals a gap between experiencing an event as meaningful and articulating that significance verbally. This recalls Peirce’s distinction between the qualitative character of feeling and its conceptual differentiation. A quality of feeling does not depend on prior analysis or comparison to be experienced (Peirce, 1931–1958, CP 1.304, 1.306). P2’s difficulty finding “the right word” should consequently not be taken as evidence that the experience lacked organization or meaning. It points to the challenge of translating the lived quality of the experience into a more specific sign.

P2 continues searching and ultimately selects the phrase “I was levitating.” The metaphor does something that a clearly differentiated emotional label might not. It gives the experience a communicable form without completely closing its meaning. “Levitating” may evoke suspension, lightness, detachment, or movement, while none of these possibilities alone exhausts what P2 appears to be trying to convey. Klempe’s (2025) analysis of the relationship between feeling and conceptual articulation is particularly pertinent here. Drawing on Kant, Klempe demonstrates that individuals may experience bodily states of pleasure and displeasure before assigning them precise conceptual forms. As previously discussed, bodily and affective experiences do not always require immediate conceptualization. What can be articulated about an experience is not identical to the entirety of what was lived. In P2’s narrative, “levitating” serves as a provisional articulation that offers greater semiotic clarity while maintaining the experience's qualitative openness.

The relationship between pleromatization and schematization becomes analytically productive at this point. It is unnecessary to infer that P2 entered a distinct “pleromatic state” before transitioning to a schematized one, as the progression can be traced within the account itself. Terms such as “integrated,” “sunk into it,” “great sound mass,” the explicit difficulty of finding “the right word,” and ultimately “levitating” illustrate various attempts to articulate an experience that remains partially captured by any single expression. Pleromatization and schematization represent distinct tendencies within this process: one preserves the expansive and affectively dense quality of experience, and the other gives aspects of it greater differentiation and communicability.

The relationship between schematization and pleromatization is not inherently oppositional. Schematization does not necessarily diminish the affective richness characteristic of pleromatization, nor does pleromatization entail a lack of semiotic organization. P2’s metaphor effectively illustrates this dynamic. The term “levitating” is more specific than the preceding hesitation, yet it retains sufficient openness to accommodate multiple qualitative possibilities. The process of moving toward articulation can maintain aspects of the experiential field from which the sign emerges.

For creativity, this relation matters because affective-semiotic organization is connected to orientation and action, not only to retrospective description. During improvisation, musicians encounter qualities that may attract, disturb, sustain, or redirect their engagement before these qualities acquire precise conceptual definitions. Some qualities become sufficiently differentiated to support a musical gesture, repetition, interruption, or change of direction, while others remain part of a broader experiential background. Pleromatization and schematization offer a way of following how creative possibilities can remain open while also cultivating enough semiotic definition to participate in subsequent action.

### Perspectival-Reflective Semiosis

Creative action does not occur from a single, fixed position. In collaborative improvisation, musicians act while responding to their own and others’ contributions, making shifts in perspective integral to the creative process. This focus on multiple perspectives underscores the inherently social nature of the mind (Vygotsky, 2007), as creators navigate shifting positional viewpoints shaped by social and cultural-historical contexts. Consistent with Mead’s relational conception of the self, perspectivism is constituted through engagement with others, material environments, and extended temporal frameworks, involving the recursive differentiation and coordination of perspectives in action (Glăveanu, 2015; Mead, 1938). In this dynamic context, perspectivization serves as a key condition for novelty, as transitions between acting and self-observation reorganize meanings and generate new possibilities for interpretation and agency (Neves-Pereira & Pinheiro, 2023).

When examined through Glăveanu’s perspectival approach, musical improvisation can be understood as more than the generation of sonic material; it is a situated and embodied process in which diverse human agents, material elements, and contextual constraints are coordinated. During improvised performance, musicians alternate between acting and perceiving, thereby positioning themselves as both contributors to and observers of the ongoing musical event (Monzo, 2025). This reflexive movement enables improvisers to continually re-evaluate the situation and orient themselves toward potential directions, fostering the emergence of novel actions and meanings within the collective activity (Glăveanu, 2021; Monzo, 2022).

These perspectival dynamics become apparent in Monzo’s (2025) empirical material, particularly in P1’s statement.

“I heard myself reacting to you… I heard you reacting to me too” (P1, Type 1 Verbal Report, Ep. 3, as cited in Monzo, 2025, p. 92).

While being interviewed, P1 further elaborated on the interactional approach he employed:

I made a lot of parallels using voices and reacted to gestures… responding, imitating. Imitating a melodic fragment or trying to respond with a retrograde. Or trying to respond with contrast, anyway, and to create textures, and dynamics together. (P1, Interview, 147–153, as cited in Monzo, 2025, p. 92).

P1’s account is significant because his contribution is not presented as an isolated decision followed by another musician’s response. The sequence is relational from the outset. He listens to his own response to the other while simultaneously attending to how the other responds to him. What he has played becomes material that can be heard and interpreted in relation to subsequent events. Although the distinction between acting and observing remains, it does not correspond to two discrete moments. P1 participates in the interaction while also encountering his participation from a shifting position within the process.

The interview gives this movement greater musical specificity. P1 describes hearing a sustained note in another musician’s playing, taking that note, placing it “on the end,” and reharmonizing it. The other musician’s gesture does not determine P1’s response. It becomes significant through P1’s listening and through musical resources developed within his own history of practice. Reharmonization renders this transformation particularly evident. The note is taken up from another musician’s contribution but becomes part of a new musical relation through P1’s action. Perspective-taking here does not mean reproducing the other musician’s perspective or recovering an original intention. It involves allowing another’s action to participate in the reorganization of one’s own.

This dynamic is also evident within the performance itself. At 3:59 and 4:02,^1^ P1 temporarily suspends sound production while P3 develops a musical idea, then returns with a transformed contribution. This suspension demonstrates that participation does not require continuous sound production. Within the framework of the 5 A’s, this moment highlights the fluidity between Actor and Audience. An improviser generating musical material may temporarily assume a more audience-like stance toward another musician’s actions, then resume sound production from a context that attentive listening has shaped. Actor and Audience are not fixed roles assigned to specific individuals; they are shifting relational positions that participants may occupy throughout the creative process.

Monzo’s material enables the tracing of this movement within a single improviser. Wilson and MacDonald (2017) reveal an additional dimension of perspectivization by comparing how different improvisers interpreted identical moments within a shared performance. Their data are particularly significant because they demonstrate that relational orientation does not necessarily result in convergence of meaning.

This episode matters not simply because the musicians remembered the event differently. Their narratives construct agency in distinct ways. The identification of who initiated the change, who responded, and the function of silence within the improvisation depends on the position from which the event is reconstructed. For 1 A, the gesture is intended to elicit a response. For 1B, the interruption creates a space generated through her own intervention. For 1 C, stopping constitutes a response, serving as a means of coordination with 1A’s actions. The same musical passage can simultaneously contribute to multiple trajectories of significance.

These findings challenge the assumption that collaborative creativity relies on musicians achieving a shared representation of ongoing events. Wilson and MacDonald (2017) identified divergent interpretations among all six trios, including disagreements regarding the initiation of musical changes and the understanding of specific gestures. Despite these differences, the musicians continued to improvise collectively. Coordination, in this case, cannot be reduced to agreement about the meaning of previous actions. What is shared is the developing situation itself, while the significance attributed to that situation can remain perspectivally differentiated.

Wilson and MacDonald (2017) further clarify the uncertainty inherent in this process. Participant 4 A describes a scenario in which the continuation of a rhythmic idea by another improviser may acquire multiple meanings:

If 4B starts the rhythm and then I join in with the rhythm then I feel in some way that I’m supporting her. If I then try and introduce a different element into it and she keeps that rhythm going there’s a chance that she’s meaning “no, listen, this is the rhythm” but there’s also the chance that “ah, that’s working, let’s stick with it.” So in that sense I never know which way that’s going: whether they’re being supportive when they change or when they stick or whatever. It’s kind of a trust thing. (4A, Wilson & MacDonald, 2017, p. 142).

In this scenario, the other’s perspective remains fundamentally unknowable. 4 A lacks access to 4B’s intention, and this ambiguity persists as 4 A must orient himself within the musical situation. Rather than resolving the uncertainty, 4 A constructs at least two possible interpretations of the same gesture. Continuing the rhythm may be perceived either as insistence on an already established direction or as confirmation that the new relational dynamic is working. Each interpretation reshapes the significance of 4B’s action for 4 A and, consequently, the options available for 4A’s subsequent actions. The final remark, “It’s kind of a trust thing,” introduces an affective dimension to this uncertainty, indicating that the willingness to act without fully understanding the other’s gesture is partly contingent on the relational context in which the ambiguity is experienced.

This is also where the embodied and affective character of perspective becomes important. Maiese’s (2014) notion of affective framing helps clarify that orientation within a situation is not simply a matter of selecting between cognitive interpretations. Affective framing participates in what becomes salient and significant and in the possibilities toward which a person becomes oriented. In 4A’s account, the two possible interpretations of 4B’s rhythmic persistence do not merely represent alternative explanations of the same event. Each organizes a different relation to her gesture. Hearing it as insistence on “the rhythm” and hearing it as confirmation that something is “working” give the event different experiential weight and potentially orient subsequent action differently. The data do not tell us which affective quality 4 A experienced, and there is no need to infer one. What they allow us to see is that perspectival uncertainty is connected to differences in how possibilities for action can become significant.

Maiese’s contribution is particularly valuable because it prevents perspectivization from being reduced to a solely cognitive process. Moving between possible perspectives engages the improviser’s entire orientation toward the situation. A possibility is not merely represented; it may assume varying degrees of relevance within an affectively framed experience. This brings perspectival-reflective semiosis back into relation with Affectivity. Shifts in perspective reorganize both the musician’s interpretation of others’ actions and the significance that potential responses assume within the course of action.

Considered together, Monzo (2025) and Wilson and MacDonald (2017) reveal complementary aspects of perspectival-reflective semiosis. According to P1’s account, an improviser alternates between producing, listening, and perceiving his own contribution through another musician’s response. Wilson and MacDonald demonstrate that such relational movement does not provide musicians with transparent access to each other’s perspectives. The same event may acquire distinct meanings for different participants, and the intentions of others often remain open to multiple interpretations. Reflexivity does not represent a progression toward a neutral vantage point from which the situation can be objectively observed. Each new position remains contextually situated and reorganizes the relationships through which the event attains meaning.

The significance of perspectivization in creativity rests on the idea that perspectives do not necessarily need to converge. Difference, ambiguity, and partial access to others may serve as generative conditions, provided that improvisers maintain the capacity to orient and reorient themselves within these dynamics. Shifts in perspective can determine what becomes salient, reshape the meaning of prior events, and reveal possibilities that were inaccessible from the previous position. Creative action emerges through such movements, as improvisers (actors) engage with shared situations from relationally constituted, yet never fully identical, positions.

### Self-Regulation in Irreversible Time

Self-regulation assumes a distinct temporal significance in the context of improvisation. After a musical gesture is executed, it becomes integrated into the evolving conditions that shape subsequent actions. The improviser is unable to revert to the prior situation, even when seeking to correct, redirect, or abandon the preceding gesture. The irreversibility of time situates regulation within a dynamic relationship between past experiences and future possibilities (Valsiner, 2014, 2021).

Monson’s (1996) analysis of an exchange between pianist Geri Allen and drummer Ralph Peterson during Allen’s solo on Peterson’s composition *Princess* provides an opportunity to examine this temporal relation. In the course of listening to a recording of the performance with Monson, Peterson reconstructs the interaction by relating Allen’s rhythmic figure to a pattern associated with Art Blakey:

“Yeah! ‘Salt Peanuts’ and ‘Looney Tunes’—kind of a combination of the two. Art Blakey has a thing he plays. […] And Geri played […] So I played the second half of the Art Blakey phrase […].” (Ralph Peterson, as cited in Monson, 1996, pp. 77).

Peterson then describes what this exchange means to him as an improvisational process:

But you see what happens is, a lot of times when you get into a musical conversation one person in the group will state an idea or the beginning of an idea and another person will complete the idea or their interpretation of the same idea, how they hear it. So the conversation happens in fragments and comes from different parts, different voices. (Ralph Peterson, as cited in Monson, 1996, p. 78).

Peterson’s analysis situates previous experience and present action in a relationship that is neither merely reproductive nor predetermined. Allen’s gesture is identifiable through a musical pattern associated with Art Blakey; however, Peterson does not simply replicate this pattern. He completes the pattern within the specific interaction where it becomes relevant. A cultural resource derived from prior musical experience is recontextualized in the present through the significance attributed to Allen’s gesture, thereby enabling a response that did not exist in advance.

Monson’s analysis of the performance introduces an additional temporal dimension. She notes that Peterson’s intervention in measures 7 and 8 may have disrupted Allen’s melodic phrase. Following this, Allen promptly presents a new and forceful idea in measure 9, which elicits another response from Peterson in measure 12 (Monson, 1996).

After Peterson responds, Allen finds herself in a different musical situation than when she started her previous phrase. Her next move now takes his reply into account, just as Peterson’s next action follows Allen’s response. This back-and-forth shifts the timing. Neither musician can return to how things were before; each must move forward from a present shaped by the other’s actions.

Sawyer’s (2006) account of group creativity shifts the focus from individual responses to the interactional dynamics through which creative action emerges. In collaborative improvisation, each person builds on what others have done, which changes what can happen next. In *Princess*, this interdependence makes the trajectory of interaction itself consequential for creative action.

A different temporal problem appears in Monson’s analysis of *Bass-ment Blues*, performed by pianist Jaki Byard, bassist George Tucker, drummer Alan Dawson, and saxophonist Joe Farrell. During Tucker’s bass solo, a discrepancy develops between his temporal position and the twelve-bar chorus structure maintained by Byard and Dawson. Monson (1996) describes the interactional problem that follows:

When Byard snaps his fingers at the beginning of what should have been chorus 4, we hear that he is maintaining the original chorus structure in his mind and is aware that there is a discrepancy between his time keeping and Tucker’s. From this moment until a consistent chorus structure is reestablished at the beginning of chorus 7, the band’s interactional challenge is to figure out how they are going to get back together […]. (Monson, 1996, pp. 157, 158).

This episode presents a different temporal problem. Previously coordinated orientations have diverged, yet the musicians cannot return to the moment before Tucker moved out of phase. “Getting back together” has to be accomplished through continued musical activity. The resulting discrepancy becomes an integral condition for reconstructing coordination.

Later in the performance, another temporal discrepancy occurs. This time, Monson describes a different response:

George Tucker gets off again by beginning a two-measure turnaround in response to Byard in measure 105, two measures earlier than its expected location. […] This time Byard and Dawson decide to go with Tucker’s turnaround: they respond by playing measure 107 as if it were the top of the chorus […].(Monson, 1996, pp. 172, 173).

In measure 107, Byard shouts ‘One!’ indicating that the band should take that measure as the top of the chorus. Tucker, however—perhaps afraid of causing a time-cycle problem again—plays a second turnaround in measures 107 and 108 […]. (Monson, 1996, p. 172).

Placed alongside the earlier disruption, this second episode shows that self-regulation cannot be reduced to the restoration of a pre-existing temporal organization. The first discrepancy requires the musicians to find a way of getting back together; when another discrepancy occurs, Byard and Dawson instead reorganize their actions around Tucker’s premature turnaround. The regulatory response is not predetermined by the type of problem encountered. It is constructed within the particular trajectory through which that problem takes form.

Valsiner’s (2014, 2021) conception of irreversible time clarifies that such moments cannot be understood merely as deviations followed by correction. Regulation occurs within an asymmetry between a consequential past and a future that has not yet assumed a fixed form. What matters, then, is not restoring a previous configuration but organizing action around conditions generated along the trajectory.

The temporal organization of self-regulation is fundamental to creativity in collaborative improvisation. Creative action involves not only the production of novel outcomes but also the reorganization of potential actions as situational conditions evolve. Each action contributes to the context from which subsequent actions emerge, resulting in creativity that unfolds through the ongoing transformation of possibilities within the interaction. Self-regulation contributes to the creative process by sustaining and redirecting action along a trajectory that remains indeterminate in advance.

### Orientation Towards an Unrepresented Future

Creative action may be oriented toward the future without necessitating a determinate representation of, forthcoming events. This distinction is especially pertinent to improvisation, in which musicians act with a forward-looking approach while the musical trajectory remains indeterminate. Such orientation does not depend on knowing what the music will become. It can take shape through possibilities that hold significance in the present without specifying their subsequent realization.

Bailey’s (1993) reflections on form in improvisation provide a particularly clear description of this prospective orientation. Discussing how improvisers relate to musical form, he writes:

“But there is a forward-looking imagination which, while mainly concerned with the moment, will prepare for later possibilities.” (Bailey, 1993, p. 111).

The distinction between preparing for possibilities and representing a specific outcome is significant. Bailey’s concept of “forward-looking imagination” focuses on present activity while also extending beyond immediate circumstances. Preparing for subsequent developments does not require specifying their exact nature. A gesture can leave certain developments available, invite them, or create conditions from which they may become musically relevant. Orientation is prospective without becoming a plan for a predetermined result.

Klempe’s (2016) discussion of expectation and ambiguity in music helps clarify this relation. Musical organization can generate expectations concerning what may follow, while the unexpected remains an important source of musical meaning. In his discussion of harmonic ambiguity, Klempe observes that expectations tend toward univocality, whereas an unexpected continuation can disturb this anticipated resolution and open the musical event to another interpretation. Expectation can consequently orient musical experience without determining the event toward which it is directed. What is anticipated establishes a possible direction, while what actually follows may confirm, transform, or depart from it.

Viram Jasani’s account of improvisation in Indian music further clarifies the relationship between orientation and openness. Jasani characterizes improvisation as the application of musical knowledge achieved through practice and listening, resulting in the creation of material that has not been predetermined:

Your improvisation comes into play when you are trying to use the information presented to you in terms of musical facts, using your ability, and the experience acquired over the years of practicing that raga, and listening to other people play that raga, to put all this together and create some new phrase or put a new idea within that raga. (Viram Jasani, as cited in Bailey, 1993, p. 9).

Jasani’s account shows that openness toward the future does not imply an absence of structure. The raga provides musical resources, and the improviser brings abilities and a history of practicing and listening. These resources orient what musicians can do without specifying the phrase they must produce. The “new phrase” is not simply retrieved from previous experience. It takes form through the musician’s engagement with possibilities made available by a culturally organized musical practice.

The affective dimension of this orientation becomes particularly visible when Jasani discusses the relation between these possibilities and the mood of the raga:

“One has to figure out a way in which the possibilities of that raga will enhance its mood.” (Viram Jasani, as cited in Bailey, 1993, p. 9).

The phrase “enhance its mood” adds an important dimension to the process. The possibilities inherent within the raga are not simply equivalent alternatives chosen mechanically by the musician. Their significance is tied to a qualitative orientation that participates in what becomes musically compelling. The musician may consequently be oriented toward a direction before the particular gesture through which that direction will be realized has taken form. Affectivity participates here in giving different weight to possibilities without transforming one of them into a predetermined outcome.

Glăveanu’s conceptualization of creative action further develops this relationship by situating action within a “horizon of possibility” that is both open and constrained by intentions, affordances, and cultural norms (Glăveanu, 2015). In improvisation, this horizon provides a field within which musicians can act toward what may follow without determining the musical form their actions will take.

For creativity, an unrepresented future is not an empty future. Possibilities within this horizon can take on different significance as creative action develops (Glăveanu, 2021). Certain possibilities become more compelling, others diminish in relevance, and new options may open up through the creative process itself. Creative orientation involves progressing toward possibilities that have gained sufficient significance to prompt action, even before their final form is determined.

## Implications

The analyses presented in this article indicate that distributed creative action is maintained not only through coordinating individuals, materials, and possibilities, but also through transformations in how these relationships cultivate significance for participants. In the examined contexts, musical materials, actions, perspectives, and possibilities do not possess a fixed experiential value. Their significance may intensify, diminish, or move as improvisers interact with each other and with the musical environment. Affectivity plays a central role in this process, shaping what becomes salient, inviting, disruptive, worthy of continuation, or possible within creative action.

This perspective extends the relational architecture of the 5 A’s by making explicit a regulatory dimension that operates across Actor, Action, Artifact, Audience, and Affordance. The proposed 6 A’s Model should consequently be understood as a heuristic extension of the 5 A’s, with Affectivity operating across its relational dimensions and not as a sixth equivalent component. The five relational dimensions identify different aspects of distributed creative activity, while Affectivity draws attention to the changing experiential significance through which relations among them become capable of sustaining, inhibiting, or redirecting action.

**Fig. 2:**
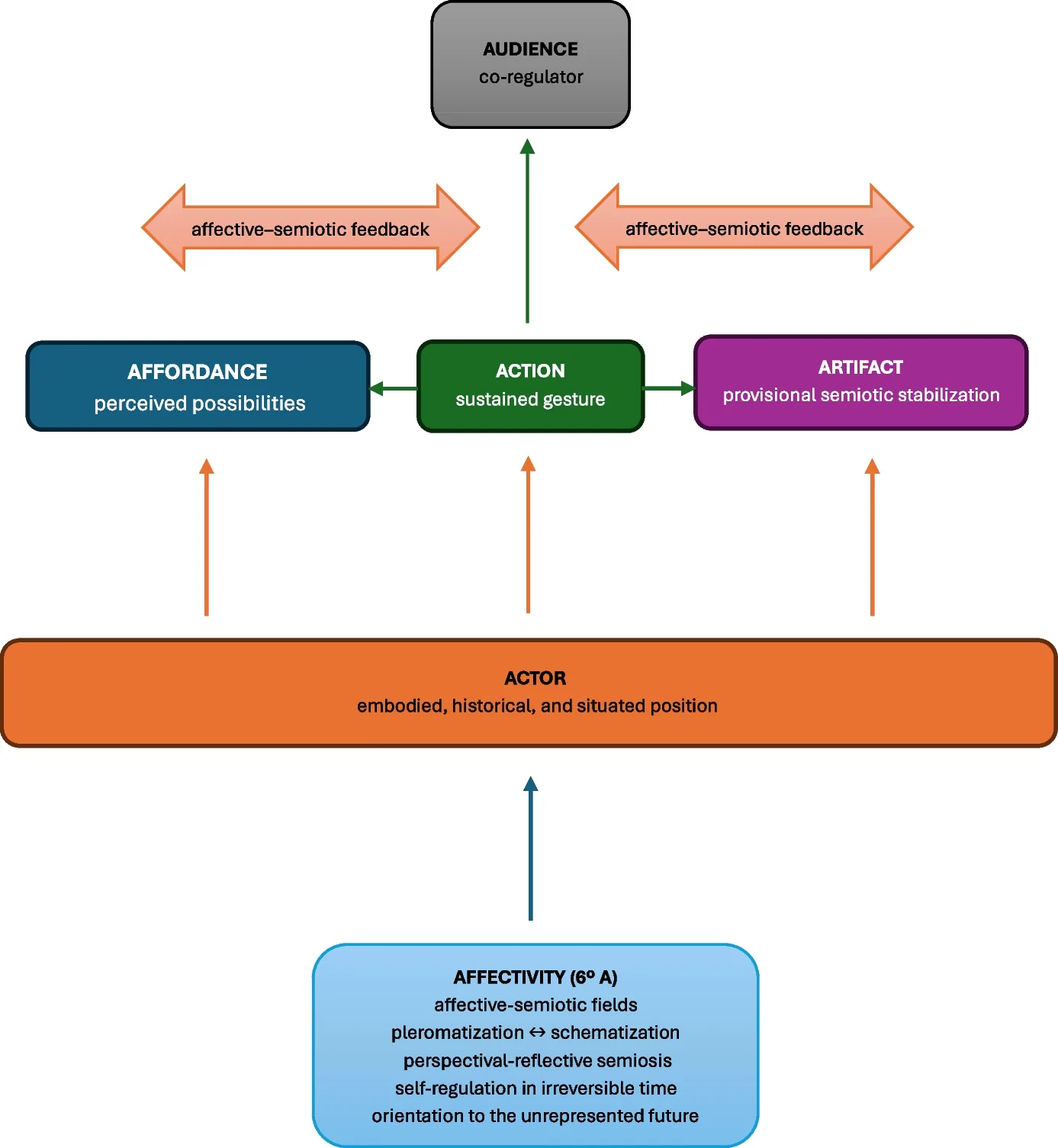
Diagram of the 6 A’s model

Figure 2 represents this transversal relation. The model redirects focus from identifying the components involved in creativity to analyzing how their interrelations gain significance within creative activity. For instance, an affordance does not regulate action just because it is available. Its regulatory relevance depends on how it is encountered within an affective-semiotic field and on the significance it achieves in relation to alternative possibilities.

The empirical materials examined here also establish limits to what can be claimed. They originate in different studies, musical practices, and methodological contexts and were not produced as a unified comparative dataset. Several of the individual studies also involve small and context-specific samples, so the selected episodes cannot establish the generality or prevalence of the proposed processes across improvisational practices. Their role in this article, however, is theoretically generative, allowing affective-semiotic processes to be examined across situations that make different aspects of creative regulation visible. The model proposed here should accordingly be treated as an analytical framework whose relations require further empirical investigation, while remaining open to empirical refinement.

Future research could examine these dynamics through research designs specifically developed to follow changes in affective-semiotic regulation across the trajectory of creative activity. Comparative studies of various improvisational practices may explore how musical conventions, material configurations, interaction histories, and levels of interpersonal familiarity impact what becomes significant for improvisers. Additionally, comparing collaborative forms involving physical co-presence with those using technological mediation or distributed improvisation could clarify how different configurations of Actor, Action, Artifact, Audience, and Affordance participate in regulating creative possibilities. Such studies may test, refine, or challenge the relationships proposed by the 6 A’s Model, while maintaining the processual nature of creativity that the model aims to capture.

## Conclusion

This article examined how an affective-semiotic perspective clarifies the regulatory dynamics that sustain and reorganize distributed creative action during musical improvisation. The analyses indicate that Affectivity is not merely an additional element alongside Actor, Action, Artifact, Audience, and Affordance; rather, it constitutes a transversal dimension through which these relationships acquire evolving significance. As musical materials, interactions, perspectives, and possibilities change throughout the improvisational process, affective-semiotic self-regulation enables the maintenance of creative activity.

Creativity depends neither on preserving stability nor on knowing in advance what will happen. It involves remaining engaged as meanings, relations, and possibilities are reorganized within the course of action. The 6 A’s Model offers a heuristic way to make this regulatory dimension visible, approaching creativity not only as the production of novelty but as the affective-sociocultural organization of possibilities through which people continue acting toward futures whose form remains open.

## Data Availability

No datasets were generated or analysed during the current study.

## Footnotes

1 https://www.youtube.com/watch?v=blDMYYsgvBI
